# Receipt of Curative Resection or Palliative Care for Hepatopancreaticobiliary Tumours (RICOCHET): Protocol for a Nationwide Collaborative Observational Study

**DOI:** 10.2196/13566

**Published:** 2019-07-08

**Authors:** The RICOCHET Study Group, West Midlands Research Collaborative

**Affiliations:** 1 Academic Department of Surgery University of Birmingham Birmingham United Kingdom

**Keywords:** ERCP, malignant jaundice, palliative, pancreatic cancer, PTC, patient pathway

## Abstract

**Background:**

There are variations in the management of patients with suspected pancreatic and periampullary cancers and/or malignant biliary obstruction. These differences may be due to a number of organizational, institutional, and patient factors that could affect outcomes for those with curable or incurable disease. The Receipt of Curative Resection or Palliative Care for Hepatopancreaticobiliary Tumours (RICOCHET) study will be the first to provide a snapshot of investigative pathways across the United Kingdom to reflect the real-world practice in these patients. The RICOCHET study is contemporary to new national and international clinical guidance and can potentially inform future local and national strategic planning to optimize care for patients with suspected hepatopancreaticobiliary (HPB) malignancies.

**Objective:**

The aim of this study is to define national variation in the investigative and management pathways of patients with suspected HPB malignancies and to determine the effect of these variations on patient outcomes.

**Methods:**

The RICOCHET study is a nationwide, multicenter, prospective study. It is led by trainees through collaboration between surgical and medical specialties. Patients with suspected pancreatic cancer, other periampullary cancer, or extrahepatic cholangiocarcinoma presenting to hospitals in the United Kingdom will be identified over 90 days. Each case will be followed up for 90 days to collect data on the mode of presentation, investigations, interventions, use of local and specialist multidisciplinary team meetings, and transfer of care between hub and spoke sites. Furthermore, the study will define dates and intervals between key points in the patient pathway.

**Results:**

The RICOCHET study results and analyses will be subject to peer review by presenting them at international cross-specialty conferences and by submitting them for publication in open-access journals. Moreover, our findings will be presented to patient groups and sponsoring charities (eg, Pancreatic Cancer UK), who in turn will disseminate key findings to the primary beneficiaries of the results: the patients. The RICOCHET study was funded in September 2017. Data collection started in April 2018 and the planned end date for data upload is spring 2019. Data analysis will take place in the summer of 2019 and the first results are expected to be published in late 2019 or early 2020.

**Conclusions:**

The RICOCHET study is a multidisciplinary, prospective, observational study that aims to highlight variability in practice and to determine whether these affect the outcomes of patients with HPB malignancies. This is a trainee-led initiative that utilizes a novel design to achieve full coverage of the differences in diagnostic and management pathways. The RICOCHET study may provide evidence to develop a more standardized approach to managing patients with suspected HPB malignancy.

**International Registered Report Identifier (IRRID):**

DERR1-10.2196/13566

## Introduction

Pancreatic cancer is the twelfth-most common cancer worldwide, but ranks fifth in its contribution to cancer-related deaths [1,2]. While the prognosis of most solid-organ cancers has improved over the last decade, the outcomes of patients with pancreatic cancer remain poor with an overall 5-year survival rate of 18% and 3.5% for patients with resectable and nonresectable disease, respectively [1,3]. This poor prognosis is partly explained by patients often presenting with advanced disease or distant metastases, due to the incipient nature by which pancreatic cancer develops and the absence of an effective screening tool [4,5]. Like pancreatic cancer, the other periampullary malignancies and extrahepatic cancers—herein collectively termed hepatopancreaticobiliary (HPB) malignancies—also have poor prognoses [6-8].

Due to the complex anatomy of the pancreas and biliary tract, the investigation of HPB malignancies requires multimodal approaches for diagnosis and staging. These tumors may involve local vascular structures and currently there is a lack of evidence regarding the optimum management of borderline and locally advanced tumors. Consequently, there is wide variability in the investigation and management of HPB malignancies between countries, but also on a national level [9,10]. It has been recognized that variability in the patient pathway can have a dramatic impact upon outcomes among these patients with regard to the time required to come to a diagnosis and referral to a specialist resectional center [11-15]. The need for better diagnostic pathways and faster access to surgery have recently been incorporated into the UK National Institute for Health and Care Excellence (NICE) guidelines for the diagnosis and management of pancreatic cancer [16]. Furthermore, the European Society for Medical Oncology recognized the rising number of deaths from pancreatic cancer in Europe and has also outlined recommendations for screening and diagnosis of pancreatic cancer [17].

In this paper, we describe the protocol of the Receipt of Curative Resection or Palliative Care for Hepatopancreaticobiliary Tumours (RICOCHET) study. This prospective study aims to define variations in diagnostic and management pathways for patients with suspected HPB malignancies, determine factors associated with these variations, and test the hypothesis that these variations have an impact on patient outcomes.

## Methods

This national, multicenter, prospective observational study will be coordinated and delivered by a cross-specialty, trainee-led research network in collaboration with surgeons, physicians, and allied health care professionals.

### Objectives

This study will define the pathways that patients with suspected HPB malignancies take from presentation to the completion of treatment, in terms of times between key diagnostic tests, multidisciplinary team (MDT) meetings, management of jaundice, treatments, and outcomes. Furthermore, it aims to define the variation in these practices and the potential effect of this variation in observed outcomes. The intention of therapy for patients with an HPB malignancy is determined by whether the primary lesion is deemed surgically resectable or not. The secondary objectives of the study are sectioned by intent of therapy (ie, resectable or nonresectable). The details of the primary and secondary objectives can be found in Table 1. Audit standards of the RICOCHET study are shown in Table 2. While patients will be analyzed by resectional status, outcomes from palliative management, which may occur concurrently, will also be collected. Therefore, referrals to a palliative care team, rates of palliative chemotherapy, and reviews by a clinical nurse specialist (CNS) have been included in our data collection.

In undertaking a cross-specialty national study, we aim to develop a lasting collaborative research network that will provide a framework for future clinical research.

**Table 1:** Primary and secondary objectives of the RICOCHET^a^ study.

Objectives and outcomes				Outcome measures
**Primary objective: To describe the management pathways and 90-day outcomes for patients who are investigated with suspected resectable and nonresectable cancer of the pancreas, periampullary tissues, and the major bile ducts**				
	**Management pathway domains**			
		Presentation to secondary care		Presentation to outpatient clinic, emergency admission, referral from spoke center, incidental radiological finding, etc
		Principal care point		Whether first presentation was at a hub or spoke center^b^
		Utility of MDT^c^		Whether case discussed at MDT meeting Timing with reference to presentation and frequency
	**Investigation domains**			
		Imaging		Timing of imaging with reference to presentation Modality and frequency USS^d^, CT^e^, MRI^f^, and PET CT^g^
		Diagnostic tissue sampling		Timing of diagnostic sampling with reference to presentation Modality and frequency EUS FNA^h^, ERCP^i^, PTC^j^ brushings, and tissue biopsy
	**Intervention domains**			
		Biliary decompression		Utility: describing indication for decompression Modality: ERCP, PTC, or other Safety: decompression modality-specific complication rates Success rates as defined by successful biliary drainage^k^
		Neoadjuvant chemotherapy		Utility: rates of use Safety: chemotherapy-specific complication rates
		Nutritional supplementation		Utility of specialist nutrition team input: rates of referral Utility of pancreatic enzyme replacement: rates of prescription
	**Intention domains**			
		Curative surgery		Time from presentation to surgery Rates of completion of surgery with curative intent or awaiting surgery Histological staging Rates of adverse events
		Palliative and end-of-life care planning		Rates of referral to hospital or community palliative care team if appropriate Number of patients seen by a CNS^l^ Number of patients where ceiling of care and resuscitation status has been discussed
		Other outcomes		Number of inpatient days Number of unplanned admissions Death: time and cause
**Secondary objectives: resectable**				
	**Subgroup comparison of patients with obstructive jaundice who undergo preoperative biliary decompression versus patients who do not have decompression**			
		Decision making		Recording reasons why biliary decompression withheld in patient with obstructive jaundice
		Management pathway, investigation, intervention, and intention domains		Comparison of outcome measures, as in primary objective, between patients who did and did not undergo biliary decompression
	**Subgroup comparison of patients who primarily attend a hub center compared to a spoke center**			
		Management pathway, investigation, intervention, and intention domains		Comparison of outcome measures, as in primary objective, between subgroups
**Secondary objectives: nonresectable**				
	**Subgroup analysis of cohort who undergo biliary decompression^k^**			
		Management pathway, investigation, intervention, and intention domains		As in primary objective, with intention to determine associations with adverse events and “other outcomes”
		Additional intention domain: palliative chemotherapy		Rates of starting palliative chemotherapy
	**Determine whether observed practice meets expected standards as defined by audit standards**			
		See Table 2		–
**Other objectives**				
	**Comparison of institutional factors and hepatobiliary services in hub and spoke centers**			
		**Institutional factors and HPB^m^** **services**		
			Hospital capacity	Number of inpatient beds
			Critical care capacity	Number of beds available for patients requiring intensive care or organ support Number of HPB surgical resections
			Interventional management of obstructive jaundice	Availability of biliary decompression services Number of decompression sessions per week
	**Assessment of data collection tools**			
		Hospital technological facilities		Proportion of sites with access to electronic reports of patient data
	**National research network development**			
		Promotion of collaborative research		Size of geographical region Number of sites in geographical regions Number of regional leads, local leads, and data collectors

^a^RICOCHET: Receipt of Curative Resection or Palliative Care for Hepatopancreaticobiliary Tumours.

^b^Hub-and-spoke design: network consisting of an anchor establishment, the hub, complemented by secondary establishments, the spokes.

^c^MDT: multidisciplinary team.

^d^USS: ultrasound scan.

^e^CT: computed tomography.

^f^MRI: magnetic resonance imaging.

^g^PET CT: positron emission tomography-computed tomography.

^h^EUS FNA: endoscopic ultrasound fine-needle aspiration.

^i^ERCP: endoscopic retrograde cholangiopancreatography.

^j^PTC: percutaneous transhepatic cholangiography.

^k^Successful decompression is defined as the successful deployment of a stent as stated on the latest procedure report.

^l^CNS: clinical nurse specialist.

^m^HPB: hepatopancreaticobiliary.

**Table 2:** Audit standards of the RICOCHET^a^ study.

Audit standard		Standard compliance, %
Patients proceeding to surgery for pancreatic cancer should be found to have metastatic disease [9]		<25
For patients undergoing first biliary decompression, stent should be placed and cytology or histology taken where appropriate [18]		>80
**Patient survival after biliary decompression in palliative disease**		
	7 days [19,20]	>90
	30 days [21]	>75
Ability to proceed to palliative chemotherapy in patients with unresectable malignancy [22]		25

^a^RICOCHET: Receipt of Curative Resection or Palliative Care for Hepatopancreaticobiliary Tumours.

### Case Identification, Inclusion, and Follow-Up

Aspects of health care in the United Kingdom are modelled on a hub-and-spoke design, which arranges service delivery assets into a network consisting of a tertiary care provider, the hub, complemented by secondary care providers, the spokes. The hub offers a specialist service, including resection, whereas the spokes offer a more limited service, routing patients needing specialist treatment to the hub [23].

**Figure 1:**
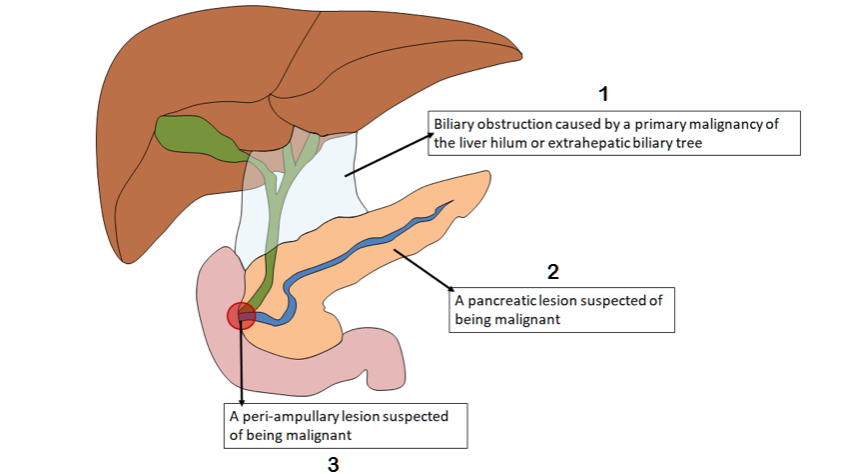
Schematic of the liver and pancreas showing the inclusion criteria for the study. To be included, patients must have one of the three indicated inclusion criteria.

**Figure 2:**
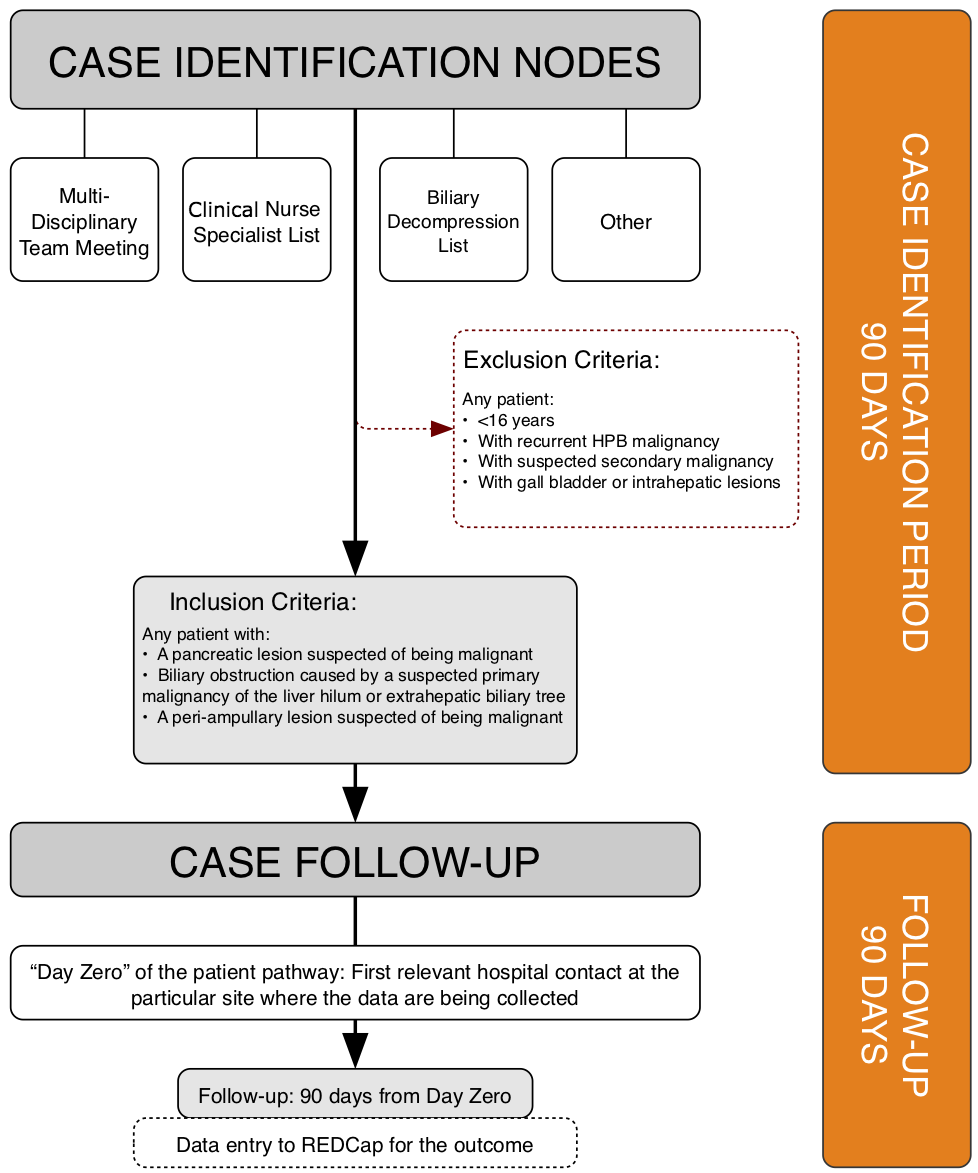
Case identification for the study. HPB: hepatopancreaticobiliary; REDCap: Research Electronic Data Capture.

Adult patients with a newly suspected HPB malignancy will be identified and screened for inclusion at participating sites over a 90-day, case-identification period. A patient will be included according to one of the three inclusion criteria: (1) suspected malignant pancreatic lesion, (2) suspected periampullary lesion, or (3) suspected malignant biliary obstruction caused by a primary malignancy of the liver hilum or extrahepatic biliary tree (see Figure 1). Exclusion criteria include the following: less than 16 years of age, recurrent HPB malignancy, suspected secondary malignancy (ie, metastatic disease of an origin outside of the HPB anatomical area), and gallbladder or intrahepatic lesions. Cases will be identified at four nodes: MDT meetings, CNS referrals, from biliary decompression lists, and any remaining modes of referral, including through outpatient clinic and ward referrals (see Figure 2). Upon inclusion to the study, each patient’s management and investigative pathways will be charted from their initial relevant presentation to hospital care at the participating site. This *day zero* will be defined as the chronologically primary relevant attendance to the emergency department, outpatient clinic, discussion of the case at MDT where a diagnosis of malignancy was first considered, or the date of the radiological or endoscopic imaging that identifies an incidental finding of malignancy. Cases will be mapped by the outcome measures described in Table 1 for 90 days from *day zero*. For cases in which a patient’s care is moved between a spoke and hub center, data will be collected from site-specific *day zero* for the following 90 days. For the purpose of analysis, we will primarily assess 90-day outcomes from the first *day zero*. However, we may explore extended time points as part of an exploratory outcome analysis. The differences in treatment and outcomes of patients across centers (ie, hub or spoke) will be analyzed.

### Sample Size

The RICOCHET study aims to involve all hub and spoke centers across the United Kingdom, and we expect to reach 75% of the cases during our inclusion period. Based on the follow-up period of 90 days and the annual incidence of the included HPB malignancies in the United Kingdom, we project the inclusion of approximately 1835 cases [1].

### Center Recruitment and Research Network

**Figure 3:**
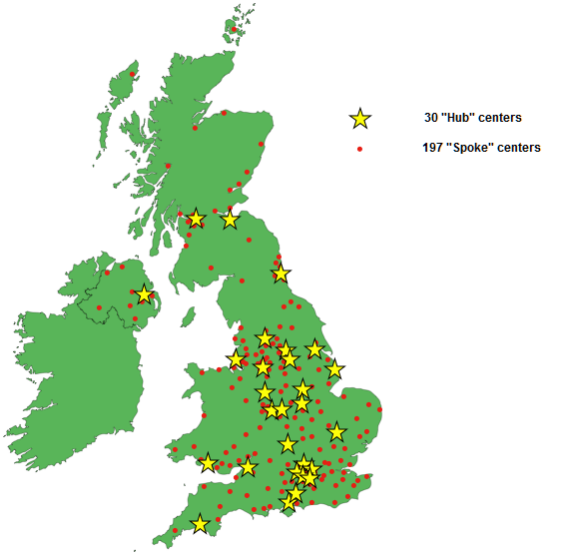
Schematic of the United Kingdom showing the location of all centers eligible for recruitment.

All centers that identify or refer HPB malignancies (N=227) are eligible to participate in this study (see Figure 3). The RICOCHET study will be open to all hub and spoke centers across the United Kingdom. Recruitment will take place via conferences, social media, established research contacts, trainee collaborative research networks, and from the use of the RICOCHET website [24].

### Project Management

**Figure 4:**
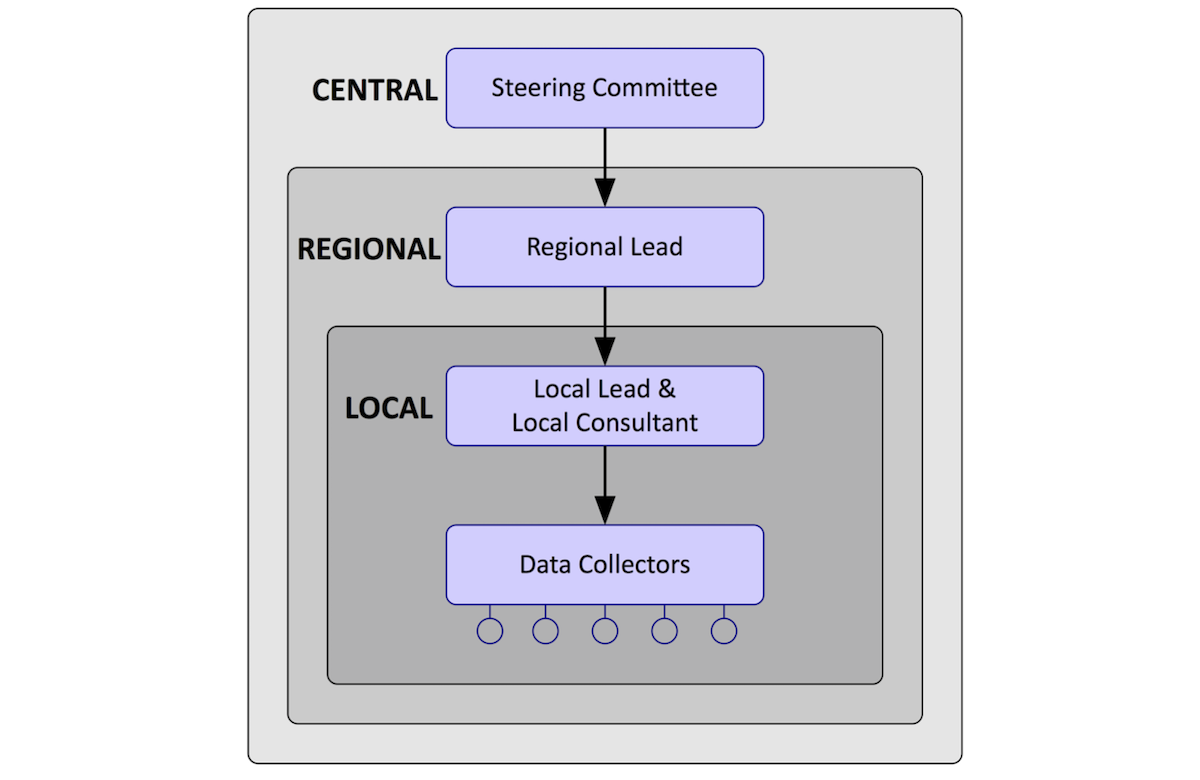
The Receipt of Curative Resection or Palliative Care for Hepatopancreaticobiliary Tumours (RICOCHET) research network.

A steering committee—formed from doctors in training alongside one medical consultant and one surgical consultant—has designed, implemented, and overseen the study as well as the analysis and dissemination of results on completion. Regional collaborators will be organized as geographical regional leads (1-2 per region; 17 regions) that will support participating sites (up to 227 sites); they consist of local consultants (1-2 per site), local leads (1 per site), and data collection teams (1-5 collectors per site) (see Figure 4). It will be encouraged that both consultant surgeons and physicians work together as part of a multidisciplinary approach. The involvement of clinical nurse specialists, research nurses, and MDT coordinators will also be encouraged. Patient representatives are involved in every step of the development of this study.

### Data Collection

Case identification and follow-up will be undertaken in the same manner at all sites. The local data collectors are either medically trained, ranging from medical students to consultants, or specialist nurses in HPB surgery or oncology. Site- and case-specific data will be entered onto Research Electronic Data Capture (REDCap), an established Web application that allows collaborators to enter and store data [25]. The REDCap server for the RICOCHET study is hosted by the Birmingham Surgical Trials Consortium, University of Birmingham, Birmingham, UK, under license from Vanderbilt University, Nashville, Tennessee. REDCap allows electronic data collection and can be accessed via a Web browser or an app on a tablet or mobile phone. REDCap has been used successfully in over 120 different countries and in more than 500,000 projects around the world [26-28].

### Data Linkage Across Sites and Pseudonymization

The RICOCHET study will involve a large number of sites across the United Kingdom to include both hub and spoke centers. Patient care may be transferred from spoke centers to specialist hub centers for discussion and/or treatment. In order to record a patient’s complete pathway, data will be collected from all sites involved in a patient’s care. However, the centers involved in the RICOCHET study have isolated, independent computer and data storage systems, with no means of centralized data access. To overcome this problem, the study will utilize a system of pseudonymization that assigns a case identifier to a patient’s REDCap records, which can be used to link REDCap data.

Data collection teams at each site will be sent the OpenPseudonymiser program via individual USB sticks. OpenPseudonymiser is a free, open-source, standalone Windows application [29] that uses the principles outlined by the Information Commissioner’s Office on data protection [30]. It has been designed to comply with national information governance requirements for the transfer of unidentifiable confidential data. OpenPseudonymiser checks the validity of the National Health Service (NHS) number within a comma-separated variables file, attaches extra encrypted data to the NHS number, then encrypts the combination using the international standard Secure Hash Algorithm 256 (SHA-256) to produce a string of output characters, known as the digest. The digest can then be used as the case identifier within REDCap. It enables data linkage across sites, as using the same NHS number with the same encryption will produce the same digest. Therefore, pseudonymization of the NHS number for each case can be achieved before being uploaded onto REDCap to maintain confidentiality of patient data and allow data linkage across sites.

A successful pilot study was carried out by the RICOCHET committee and involved testing of the REDCap and pseudonymization systems to allow accurate patient data linkage across hub and spoke sites before being rolled out nationally.

### Statistical Analysis

Upon data collection and dissemination, data distribution will be determined and appropriately summarized. Frequencies and percentages will be used for categorical variables. Univariate and multivariate analyses will be assessed by appropriate statistical techniques. A *P* value of less than .05 will be considered significant for statistical methods used. The analysis will be completed by suitable statistical software.

### Ethics, Consent to Participate, and Dissemination

The RICOCHET study is a prospective study mapping patient investigative and management pathways. An intervention involving the patient’s health care will not be implemented; therefore, patient consent is not required for the RICOCHET study. This has been confirmed using the national UK decision-making tool of the NHS Health Research Authority and the Medical Research Council [31]. The RICOCHET study will therefore be locally registered as a clinical audit or service evaluation project at all participating sites prior to patient identification and data collection.

### Availability of Data and Material

The datasets used and/or analyzed during the current study are available from the corresponding author upon reasonable request.

## Results

The RICOCHET study results and analyses will be subject to peer review by presenting them at international cross-specialty conferences and by submitting them for publication in open-access journals. Moreover, our findings will be presented to patient groups and sponsoring charities (eg, Pancreatic Cancer UK), who in turn will disseminate key findings to the primary beneficiaries of the results: the patients. The RICOCHET study was funded in September 2017. Data collection started in April 2018 and the planned end date for data upload is spring 2019. Data analysis will take place in the summer of 2019 and the first results are expected to be published in late 2019 or early 2020.

## Discussion

Survival among patients with pancreatic cancer has not improved over the past 40 years, a fact that demands the attention of health care providers, users, and service designers [32,33]. Nevertheless, there are grounds for optimism. Adjuvant chemotherapy is increasingly effective, correction of pancreatic exocrine insufficiency can improve survival, and optimized diagnostic pathways can reduce the time to surgery and improve resection rates [9,34,35]. These are just a few examples of where progress is being made. Optimizing pathways, reducing variation in practice, and national guidance can all help achieve improvement. While more substantial improvements may occur from novel chemotherapeutics, it is clear that outcomes and patient experience can be improved by focusing on optimizing every part of the patient pathway, from diagnosis to treatment [36]. It is therefore essential that current practice and its variation and effect on patient outcomes are evaluated. This study closely follows the publication of the first NICE guidelines for the management of pancreatic cancer in the United Kingdom [16]. Real-world practice may stray from guidelines for a multitude of reasons, including limitations of local resources and expertise, case-specific vagaries, and, in some cases, perceived equipoise in the available data [9]. There may also be a tendency to overinvestigate patients, with consequent delays to treatment [10]. The RICOCHET study aims to reveal the main bottlenecks in the pathways and identify where improvements can be made.

The 15% of patients with potentially resectable disease are frequently the focus of clinical research, with a limited emphasis on the considerably larger proportion of patients with nonresectable disease [37]. The RICOCHET study is a comprehensive study of practice among all patients with suspected cancer, regardless of stage or treatment options. Analysis of Hospital Episode Statistics data demonstrates a remarkably high 30-day mortality rate among patients with malignant biliary obstruction, but the cause for this high incidence remains unknown [19]. A further benefit of the RICOCHET study is, therefore, to target areas where there is a particular need for more in-depth information.

Collecting patient-level data across hospitals with linkage presents significant ethical challenges. The use of the OpenPseudonymiser tool to link patient data across independent sites overcomes this potential prohibitive barrier to patient-pathway mapping. Successful implementation of this system in an ambitious nationwide study will provide a blueprint for future collaborative research that requires linking patient data from discrete sites.

The RICOCHET study has several limitations. The study has been designed to assess patient pathways against contemporary guidelines [16,17]. The 90-day patient follow-up period reflects this, but denies the assessment of medium- and long-term outcomes. We expect that the data gathered by the RICOCHET study will inform focused cohort studies and randomized controlled trials that are designed to comprehensively answer questions about medium- and long-term outcomes. Furthermore, the nature of this observational study precludes an assessment of quality of life and patient-reported outcomes; these are critical in the meaningful assessment of care in patients with cancer, resectable or otherwise [38]. Over the course of the RICOCHET study, we aim to involve more than 500 collaborators across specialties, creating an extensive network of enthusiastic individuals. It will build a strong foundation for future collaborative research and strengthen interest in improving patient care in the NHS and beyond.

In conclusion, the RICOCHET study is an ambitious, multidisciplinary, multicenter, prospective observational study utilizing a novel design to achieve full coverage of the different patient pathways. It is led by trainees and builds on an extensive national collaborative network. The study aims to highlight the variation in practice and its effect on the outcomes of patients with HPB malignancies. It may then provide evidence to develop a more standardized approach to managing patients with suspected HPB malignancy.

## Abbreviations

CNS: clinical nurse specialist
CT: computed tomography
ERCP: endoscopic retrograde cholangiopancreatography
EUS FNA: endoscopic ultrasound fine-needle aspiration
HPB: hepatopancreaticobiliary
MDT: multidisciplinary team
MRI: magnetic resonance imaging
NHS: National Health Service
NICE: National Institute for Health and Care Excellence
PET CT: positron emission tomography-computed tomography
PTC: percutaneous transhepatic cholangiography
REDCap: Research Electronic Data Capture
RICOCHET: Receipt of Curative Resection or Palliative Care for Hepatopancreaticobiliary Tumours
SHA-256: Secure Hash Algorithm 256
USS: ultrasound scan

## References

[1] (2018). Cancer Research UK.

[2] Malvezzi M, Bertuccio P, Levi F, La Vecchia C, Negri E (2014). European cancer mortality predictions for the year 2014. Ann Oncol.

[3] Schnelldorfer T, Ware AL, Sarr MG, Smyrk TC, Zhang L, Qin R, Gullerud RE, Donohue JH, Nagorney DM, Farnell MB (2008). Long-term survival after pancreatoduodenectomy for pancreatic adenocarcinoma: Is cure possible?. Ann Surg.

[4] Kamisawa T, Isawa T, Koike M, Tsuruta K, Okamoto A (1995). Hematogenous metastases of pancreatic ductal carcinoma. Pancreas.

[5] Disibio G, French SW (2008). Metastatic patterns of cancers: Results from a large autopsy study. Arch Pathol Lab Med.

[6] DeOliveira ML, Cunningham SC, Cameron JL, Kamangar F, Winter JM, Lillemoe KD, Choti MA, Yeo CJ, Schulick RD (2007). Cholangiocarcinoma: Thirty-one-year experience with 564 patients at a single institution. Ann Surg.

[7] Rea DJ, Heimbach JK, Rosen CB, Haddock MG, Alberts SR, Kremers WK, Gores GJ, Nagorney DM (2005). Liver transplantation with neoadjuvant chemoradiation is more effective than resection for hilar cholangiocarcinoma. Ann Surg.

[8] Yeo CJ, Sohn TA, Cameron JL, Hruban RH, Lillemoe KD, Pitt HA (1998). Periampullary adenocarcinoma: Analysis of 5-year survivors. Ann Surg.

[9] Roberts KJ, Prasad P, Steele Y, Marcon F, Faulkner T, Cilliers H, Dasari B, Abradelo M, Marudanayagam R, Sutcliffe RP, Muiesan P, Mirza DF, Isaac J (2017). A reduced time to surgery within a 'fast track' pathway for periampullary malignancy is associated with an increased rate of pancreatoduodenectomy. HPB (Oxford).

[10] Driedger MR, Dixon E, Mohamed R, Sutherland FR, Bathe OF, Ball CG (2015). The diagnostic pathway for solid pancreatic neoplasms: Are we applying too many tests?. J Surg Res.

[11] Moole H, Bechtold M, Puli S (2016). Efficacy of preoperative biliary drainage in malignant obstructive jaundice: A meta-analysis and systematic review. World J Surg Oncol.

[12] Sanjeevi S, Ivanics T, Lundell L, Kartalis N, Andrén-Sandberg Å, Blomberg J, Del Chiaro M, Ansorge C (2016). Impact of delay between imaging and treatment in patients with potentially curable pancreatic cancer. Br J Surg.

[13] Raman SP, Reddy S, Weiss MJ, Manos LL, Cameron JL, Zheng L, Herman JM, Hruban RH, Fishman EK, Wolfgang CL (2015). Impact of the time interval between MDCT imaging and surgery on the accuracy of identifying metastatic disease in patients with pancreatic cancer. AJR Am J Roentgenol.

[14] Glant JA, Waters JA, House MG, Zyromski NJ, Nakeeb A, Pitt HA, Lillemoe KD, Schmidt CM (2011). Does the interval from imaging to operation affect the rate of unanticipated metastasis encountered during operation for pancreatic adenocarcinoma?. Surgery.

[15] van der Gaag NA, Rauws EA, van Eijck CH, Bruno MJ, van der Harst E, Kubben FJ, Gerritsen JJ, Greve JW, Gerhards MF, de Hingh IH, Klinkenbijl JH, Nio CY, de Castro SM, Busch OR, van Gulik TM, Bossuyt PM, Gouma DJ (2010). Preoperative biliary drainage for cancer of the head of the pancreas. N Engl J Med.

[16] (2018). Pancreatic Cancer in Adults: Diagnosis and Management.

[17] Ducreux M, Cuhna AS, Caramella C, Hollebecque A, Burtin P, Goéré D, Seufferlein T, Haustermans K, Van Laethem JL, Conroy T, Arnold D, ESMO Guidelines Committee (2015). Cancer of the pancreas: ESMO Clinical Practice Guidelines for diagnosis, treatment and follow-up. Ann Oncol.

[18] ERCP Working Party (2014). ERCP–The Way Forward: A Standards Framework.

[19] Harvey P, Baldwin S, Mytton J, Coupland B, Evison F, Patel P, Trudgill N (2018). PTH-032 Mortality following ERCP for benign pathology in England between 2003 and 2015. Gut.

[20] Rees J, Mytton J, Evison F, Patel P, Trudgill N (2016). OC-075 Outcomes of percutaneous transhepatic cholangiography for the palliative relief of malignant jaundice in England between 2001 and 2014. Gut.

[21] (2019). NHS Digital.

[22] Harvey P, Baldwin S, Mytton J, Evison F, Patel P, Trudgill N (2017). OC-002 The outcomes of ERCP for the palliation of malignant jaundice in england between 2001 and 2014. Gut.

[23] Elrod J, Fortenberry J (2017). The hub-and-spoke organization design: An avenue for serving patients well. BMC Health Serv Res.

[24] The RICOCHET Study.

[25] REDCap.

[26] Møller P, Olling K, Berg M, Habæk I, Haislund B, Iversen AM, Ewertz M, Lorenzen E, Brink C (2018). Breast cancer patients report reduced sensitivity and pain using a barrier film during radiotherapy: A Danish intra-patient randomized multicentre study. Tech Innov Patient Support Radiat Oncol.

[27] Gonzalez DO, Lawrence AE, Cooper JN, Sola R Jr, Garvey E, Weber BC, St Peter SD, Ostlie DJ, Kohler JE, Leys CM, Deans KJ, Minneci PC (2018). Can ultrasound reliably identify complicated appendicitis in children?. J Surg Res.

[28] RIFT Study Group on behalf of the West Midlands Research Collaborative (2018). Right Iliac Fossa Pain Treatment (RIFT) Study: Protocol for an international, multicentre, prospective observational study. BMJ Open.

[29] OpenPseudonymiser.

[30] (2018). Guide to the General Data Protection Regulation (GDPR).

[31] (2017). Health Research Authority Decision Tools.

[32] Quaresma M, Coleman M, Rachet B (2015). 40-year trends in an index of survival for all cancers combined and survival adjusted for age and sex for each cancer in England and Wales, 1971-2011: A population-based study. Lancet.

[33] All Party Parliamentary Group on Pancreatic Cancer (2013). Time to Change the Story: A Plan of Action for Pancreatic Cancer.

[34] Neoptolemos J, Palmer D, Ghaneh P, Psarelli E, Valle J, Halloran C, Faluyi O, O'Reilly DA, Cunningham D, Wadsley J, Darby S, Meyer T, Gillmore R, Anthoney A, Lind P, Glimelius B, Falk S, Izbicki J, Middleton GW, Cummins S, Ross PJ, Wasan H, McDonald A, Crosby T, Ma YT, Patel K, Sherriff D, Soomal R, Borg D, Sothi S, Hammel P, Hackert T, Jackson R, Büchler MW, European Study Group for Pancreatic Cancer (2017). Comparison of adjuvant gemcitabine and capecitabine with gemcitabine monotherapy in patients with resected pancreatic cancer (ESPAC-4): A multicentre, open-label, randomised, phase 3 trial. Lancet.

[35] Roberts K, Schrem H, Hodson J, Angelico R, Dasari B, Coldham C, Marudanayagam R, Sutcliffe R, Muiesan P, Isaac J, Mirza D (2017). Pancreas exocrine replacement therapy is associated with increased survival following pancreatoduodenectomy for periampullary malignancy. HPB (Oxford).

[36] Roberts K (2017). Improving outcomes in patients with resectable pancreatic cancer. Br J Surg.

[37] Toesca D, Koong A, Poultsides GA, Visser B, Haraldsdottir S, Koong A, Chang D (2018). Management of borderline resectable pancreatic cancer. Int J Radiat Oncol Biol Phys.

[38] (2019). NHS Digital.

